# Strategic establishment of an International Pharmacology Specialty Laboratory in a resource-limited setting

**DOI:** 10.4102/ajlm.v7i1.659

**Published:** 2018-02-12

**Authors:** Takudzwa J. Mtisi, Charles Maponga, Tsitsi G. Monera-Penduka, Tinashe Mudzviti, Dexter Chagwena, Faithful Makita-Chingombe, Robin DiFranchesco, Gene D. Morse

**Affiliations:** 1 International Pharmacology Specialty Laboratory, School of Pharmacy, University of Zimbabwe College of Health Sciences, Harare, Zimbabawe; 2 Center for Integrated Global Biomedical Sciences, University at Buffalo, Buffalo, New York, United States

## Abstract

**Background:**

A growing number of drug development studies that include pharmacokinetic evaluations are conducted in regions lacking a specialised pharmacology laboratory. This necessitated the development of an International Pharmacology Specialty Laboratory (IPSL) in Zimbabwe.

**Objectives:**

The aim of this article is to describe the development of an IPSL in Zimbabwe.

**Methods:**

The IPSL was developed collaboratively by the University of Zimbabwe and the University at Buffalo Center for Integrated Global Biomedical Sciences. Key stages included infrastructure development, establishment of quality management systems and collaborative mentorship in clinical pharmacology study design and chromatographic assay development and validation.

**Results:**

Two high performance liquid chromatography instruments were donated by an instrument manufacturer and a contract research organisation. Laboratory space was acquired through association with the Zimbabwe national drug regulatory authority. Operational policies, standard operating procedures and a document control system were established. Scientists and technicians were trained in aspects relevant to IPSL operations. A high-performance liquid chromatography method for nevirapine was developed with the guidance of the Clinical Pharmacology Quality Assurance programme and approved by the assay method review programme. The University of Zimbabwe IPSL is engaged with the United States National Institute of Allergy and Infectious Diseases Division of AIDS research networks and is poised to begin drug assays and pharmacokinetic analyses.

**Conclusions:**

An IPSL has been successfully established in a resource-limited setting through the efforts of an external partnership providing technical guidance and motivated internal faculty and staff. Strategic partnerships were beneficial in navigating challenges leading to laboratory development and training new investigators. The IPSL is now engaged in clinical pharmacology research.

## Introduction

Ideally, research institutes should have access to a full complement of analytical laboratories within their jurisdiction. The laboratories should be well equipped and staffed with competent analytical scientists. Since many clinical studies involve drug interventions and pharmacokinetic evaluations, accessible assays should include analytical methods for common approved and investigational therapeutic interventions. This enables the laboratory to serve a wide range of clinical trial protocols.

There has been a growing number of clinical studies involving prevention and therapeutic drug interventions conducted in Zimbabwe. Most of the studies are conducted by the University of Zimbabwe (UZ) College of Health Sciences’ Clinical Trials Unit (UZCHS-CTU) through research networks sponsored by the United States’ National Institutes of Health (NIH). Currently the UZCHS-CTU collaborates with five HIV research networks, including the AIDS Clinical Trials Group (ACTG), HIV Prevention Trials Network, International Maternal Pediatric Adolescent AIDS Clinical Trials, Microbial Trials Network and HIV Vaccines Trials Network.^1^ Within the ACTG structure, specialties in immunology, virology, pharmacology, tuberculosis and genomics form the ACTG Laboratory Center Network structure. Prior to establishment of the UZ International Pharmacology Specialty Laboratory (IPSL), the UZ-University of California, San Francisco CTU had capacities in the other ACTG HIV laboratory specialties except pharmacology. Pharmacology specialty laboratories (PSLs) lead drug development research projects that include bioavailability, drug-drug interactions and determining pharmacokinetic parameters for drugs. The UZCHS-CTU implements Phase II and III protocols involving numerous licensed antiretroviral and other new investigational drugs. In addition, the use of traditional medicines in Zimbabwe and their potential drug interactions^2^ as well as other culture-specific issues, for example dietary habits,^3^ create a need for building pharmacology research capacities. While the laboratory is currently developing expertise in the assaying of drug concentrations in biological matrices, current research protocol requires that these samples be transported to other countries for analysis.

Shipping research biological specimens across national borders impacts clinical research processes negatively. The shipment approval procedures to assure ethical analysis of the samples are laborious, time-consuming, costly and have resulted in abortion of clinical studies in the past.^4^ National institutional review boards are also more rigorous when reviewing such protocols, as there is need to assure conformance on the part of laws of different countries. The ethical review process, which has to be as explicit as possible on the plans for the samples, is consequently more complicated and time-consuming. In addition, there is often a hesitation from local participants to enrol in studies that involve shipment of samples to other countries. The time taken with sample shipment procedures and transportation delays research timelines. This is usually accompanied by high shipping charges, which increase the cost of research. In addition, the integrity of samples may be compromised, if shipping procedures are not followed correctly as they are moved long distances across borders. Lastly, ensuring that all analytical procedures conform to what has been established during the informed consent process becomes more complicated across jurisdictions. The major challenge is that it is often more difficult for originating institutional review boards to conduct inspections of the recipient laboratories against their intended expectations.^5^

In 2007, UZ and the University at Buffalo (UB), State University of New York, United States, entered into a memorandum of understanding; its scope included teaching, research, exchange of faculty and students, as well as staff development. This formed on the backbone of the initial UB-UZ initiative that has been active since 2002. In 2005, a formal agreement to address clinical pharmacology research and training needs was adopted. Consequently, a progressive series of awards and milestones enabled UB to guide UZ during the establishment of the IPSL in Zimbabwe. This manuscript describes the collaborative development process, including challenges and strategies.

## Development and implementation process

### Competitive research supporting awards as a foundation for the International Pharmacology Specialty Laboratory

Multiple funding sources were utilised to develop and implement the UZ-IPSL. In 1998, the ACTG sponsored a fellowship in Clinical Pharmacology at UB that was completed by the current UZ-IPSL Principal Investigator. In 2009, the ACTG Principal Investigator and the Co-Principal Investigator visited UZ to discuss and assess the potential for infrastructure and subsequent capacity building to develop, implement and operate an IPSL. Shortly after this visit, the University of California, Berkley AIDS International Research and Training Program provided a sub-award to UB. This award supported two Master of Philosophy candidates from the UZ School of Pharmacy and Department of Pharmacology and created a seed that would lead to an AIDS International Research and Training Program (AITRP) focused on clinical pharmacology research between UZ and UB (2009) and begin the UZ-IPSL investigator training. The NIH Division of AIDS (DAIDS) increased the scope of the HIV Research Network’s pharmacology laboratory network to include the UZCHS IPSL, enabling the DAIDS Clinical Pharmacology Quality Assurance Program (CPQA) to assist in this process.^6^ Finally, in December 2011, the UZ-IPSL successfully competed for a Developmental PSL that would interact with each of the HIV Research Networks.

### Infrastructure and equipment

Initially, laboratory space was allocated within the University of Zimbabwe. Laboratory equipment donations were solicited from manufacturers through UB. Donations from Waters Associates, Inc., Research Triangle International and Merck Laboratories included two high-performance liquid chromatography (HPLC) systems, basic laboratory equipment, laboratory supplies and computer software for pharmacokinetic analysis. Additionally, basic equipment such as refrigerators, freezers, centrifuges, pH meters and evaporators were purchased through the NIH UZ-IPSL developmental specialty laboratory grant award. The Scientific and Industrial Research and Development Centre provided the required certification and calibration of ancillary equipment. With the addition of two HPLC systems, an agreement was made in 2012 with the Ministry of Health and Child Care through a partnership with the national drug regulatory authority and the Medicines Control Authority of Zimbabwe (MCAZ), to house the laboratory at the MCAZ. To ensure completion of assays without interruption, integrity of stored specimens and protection of sensitive laboratory equipment, a backup generator and uninterrupted power supply systems were installed.

### Human resources development

The roles within the UZ-IPSL were distributed among a team of laboratory scientists, technologists and AITRP fellows. Table 1 provides an overview of the team’s established positions, the diverse professional backgrounds, and training received to support the development of this laboratory research team. Training focused on the development of chromatographic methods, laboratory quality management systems (QMS) and Clinical Pharmacology research and most was done at the Translational Pharmacology Research Core (TPRC) at UB, New York, United States. Additional technical training was done in-house through the CPQA programme.^6,7^ In the process, individual training records were documented to provide evidence to support the appropriateness of appointed responsibilities in the various areas of research shown in Table 1.

**TABLE 1:** UZ-IPSL positions, professional qualifications and training received.

Laboratory position (initial support)	Development phase			Since 2003/MOU
	Professional qualifications (initial)	Training received† (during)	Academic qualifications (obtained after)	Current status
Laboratory Principal Investigator(ACTG & AITRP)	PharmD; MPHE; Associate Professor	ACTG Fellowship in Clinical Pharmacology	n/a	Associate Professor
Quality Management Systems Implementation Manager (AITRP & ACTG)	HBMLS	1–10 AITRP	MSc	DPhil candidate
Laboratory Supervisor (AITRP & ACTG)	BSc, Applied Biology and Biochemistry	1–10 AITRP CPQA	Diploma in Business Administration	MPhil candidate
Technical Staff (ACTG)	Certificate Chemistry National Diploma Chemistry Medical Laboratory Management	1,2,3,4,7,9,10CPQA	i) Diploma Chemistry	Laboratory Intern Laboratory Technician Resources and Facilities Implementation Technician
New Investigators/Scientists (AITRP)	BPharm BPharm, MPhil BSc Nutrition	1,2,3,4,7,9,10CPQA	MPhil MPhil –	DPhil DPhil candidate MPhil candidate

† Key for training:
Human health research (CITI) Responsible conduct of research (CITI) Adhering to confidentiality standards (CITI) Chemical and biohazard laboratory safety (In-house TPRC/UZ) International and national shipping policies: Shipping of dangerous goods (IATA) Quantitative analysis and laboratory mathematics (TPRC) Laboratory data management software training (CPQA/Frontier Science) Quality management systems and auditing (SADCAS/CPQA) Equipment specific setup and maintenance (TPRC)
General equipment Chromatography specific equipment Method development and validation (TPRC)

One laboratory scientist, the QMS implementation manager, was tasked with developing the standard operating procedures (SOPs) and overseeing the implementation of the QMS. The role involved conducting onsite monitoring and mentoring in clinical laboratory sciences. A second laboratory scientist, the laboratory supervisor, was tasked with implementing the QMS system and assay methods, as well as technician training. Both were trained extensively on chromatographic assays at the National Institute of Allergy and Infectious Diseases laboratory data management system. One laboratory technician was tasked with ensuring efficient resource utilisation, facilities improvement and maintenance. Two technicians worked under the direct supervision of the laboratory supervisor.

When the UZ-IPSL received initial designation as a Developmental PSL in 2011, it was utilised as a core laboratory to support concurrently running capacity building programmes. In this role, the UZ-IPSL provided bioanalytical training support to AITRP fellow research projects and DAIDS HIV Research Network protocols, through assay planning, mentoring and seminars in clinical pharmacokinetics and pharmacodynamics for research protocol design and data analysis.

### High-performance liquid chromatography method development and validation

HPLC methods for the determination of antiretroviral drugs in human plasma were developed under the technical guidance of CPQA. Initial assay development and validation focused on the determination of nevirapine in plasma. Nevirapine was considered highly relevant to the UZ-IPSL development agenda, because it was widely used in the setting. Subsequently, an HPLC method for efavirenz was developed and validated.

### Policies and standard operating procedures

Using the ISO 15189:2009 standard for medical laboratories, SOPs were developed for all laboratory processes.^8^ More than 30 SOPs were developed. The SOPs were used to validate all the equipment used to develop and validate HPLC methods, to ensure equipment performance in the Zimbabwean setting. ISO 15189 was specifically chosen as it covers requirements that would need to be addressed to achieve the laboratory’s vision of enlarging the scope of the laboratory to include performing pharmacology tests for clinical decision-making. The aim was to target accreditation with Southern African Development Community Accreditation Service (SADCAS), a regional, non-profit, multi-economy accreditation body whose mission is to provide credible, cost-effective accreditation services. Under the twinning partnership agreement between SADCAS and the South African Nation Accreditation System, it is now mandatory that all medical laboratories performing clinical assays in Zimbabwe seeking to obtain ISO 15189 accreditation go through the SADCAS.^9^

Oversight for quality assurance of both standard and novel assays was provided by the CPQA Proficiency Testing programme.^6^ Most clinical studies conducted in Zimbabwe are NIH funded so meeting CPQA requirements was strategic. The process was guided by the CPQA and included individual staff development, mentored laboratory training, continuous technical guidance and communications and site assessments to monitor progress. An initial site development assessment was done in 2011 from which the CPQA made specific infrastructure and QMS development recommendations. To meet CPQA requirements, the UZ-IPSL performed assays on proficiency testing samples, generated assay validation report reviews, and participated in several DAIDS cross-network clinical trial and clinical pharmacology laboratory group networking activities. Progress was tracked electronically and through continued interaction between CPQA and UZ-IPSL personnel. The most recent assessment, an implementation assessment, was done in 2016 and was the basis for the recommendation for ISO accreditation.

## Success parameters

We set out to develop a laboratory that would be acceptable in Zimbabwe with the initial primary focus to support the ACTG scientific agenda by participating in ACTG protocols. As this involved bringing together professionals from different scientific disciplines, different levels of training were required and tailored to each individual. Essentially, investigators obtained training in the order of basic laboratory, pharmacology, then chromatography training. Once these fundamentals were achieved, the laboratory then embarked on and successfully completed the method development and validation required to analyse ACTG protocol samples following CPQA approval.

Evidence of productivity associated with the UZ-IPSL can be highlighted by the increasing number of funded grants, manuscripts and collaborations throughout the development process. The UB-UZ AITRP was recently funded for another five years; it is now known as the UB-UZ HIV Research Training Program and has since been awarded additional supplemental grants in Oncology and Behavioral Sciences. One investigator also received a Center for AIDS Research award through the University of Rochester to support doctoral research. Other fellows have also received scholarship and fellowship awards from various sponsors including the Letten Foundation, World Health Organization Special Programme for Research and Training in Tropical Diseases Career Development Fellowships and the UZ Promoting Excellence in Research and Faculty Enhanced Career Training programme. In addition, from 2008 to date, 20 manuscripts have been written and numerous abstracts and conference presentations made by UZ-IPSL investigators and fellows at various international conferences including those organised by the African Society for Laboratory Medicine, the International AIDS Society and the Society of Quality Assurance.

## Discussion

Overall, implementation of the strategy was successful. However, it was not without challenges.

First, the CPQA requirements and SADCAS accreditation requirements had to be met. In contrast to laboratories conducting routine clinical chemistry tests, CPQA developmental and implementation assessments are necessary for PSLs seeking to offer pharmacology assay services to ACTG-funded studies. Once the laboratory was operational, methods required CPQA approval and then proficiency testing participation to maintain assurance of continuous competency. In order to expand the scope to offer accredited clinical laboratory services, local regulations require all clinical medical laboratories to receive relevant accreditation (in this case ISO 15189) through SADCAS. Aiming to achieve both simultaneously demanded more time, effort and resources. As a strategy, development of SOPs focused on meeting the rigorous ISO 15189 requirements. However, ISO 15189 standards are particular for medical laboratories and do not address some of the intricacies of pharmacology. Therefore, the detail of the CPQA-recommended policies and procedures were then incorporated. CPQA approval for the first UZ-IPSL HPLC method, *Determination of Nevirapine in Human Plasma*, was officially attained in December 2016. This gave the UZ-IPSL an opportunity to contribute to pharmacology assay services for international protocols, and secure additional funding to proceed with addressing the outstanding ISO 15189 requirements.

Another ongoing challenge has been the recruitment and retention of laboratory scientists with adequate training to perform drug assays. Two important criteria need to be fulfilled by suitable scientists to work in a PSL: the ability to work with biological matrices and an understanding of clinical pharmacology assays. There are currently only two programmes in the country training medical laboratory scientists and neither programme has a strong clinical pharmacology component. As such, extra training in clinical pharmacology is required. While the UZ-PSL development process has successfully trained two medical laboratory scientists, retaining them before the laboratory secures substantial assay contracts has been a challenge. As a retention strategy, only one medical laboratory scientist was contracted on a full-time basis with a competitive salary. The other was contracted on a part-time basis and prioritised for postgraduate training support from the AITRP. The AITRP also provided mentorship for laboratory-based studies, enabling the second laboratory scientist to work and study in the same environment while applying the skills to a specific AITRP project. This arrangement was a very effective retention strategy and has served to enhance the outcomes for both the funded programmes.

Securing service contracts for the HPLC instrumentation was also a challenge. There are currently limited technical support and supplies available in Zimbabwe due to the prevailing socioeconomic situation. This lack of expedient supply chains for instrument engineers threatened to slow down laboratory development. The UZ-IPSL had to rely on regional vendors, further constraining the limited laboratory funding. Close liaison with an established regional IPSL and the CPQA programme has assisted the UZ-IPSL in locating cost-effective regional vendors. In addition, a new HPLC system was purchased through ACTG support to sustain current analytical capacity.

## Future goals and challenges

The collaboration of the UZ-IPSL with the UZCHS-CTU will ensure that investigators within the network, who themselves may have other research or clinical studies, will continue to utilise and hence sustain the UZ-IPSL’s research agenda. In addition, a newly funded HIV Research and Training Program will continue to utilise the UZ-IPSL as a foundational resource to support research projects for its doctoral students and postdoctoral fellows. These fellows have assumed scientific leadership responsibility in multiple research areas including bioequivalence, nanomedicine, infectious diseases, cancer, translational pharmacology, pharmacovigilance, nutrition pharmacology, phytopharmacology and pharmacogenomics within the UZ-IPSL. This will lead to growth in the types of drug assays that are available in the UZ-IPSL to support DAIDS network clinical trials, as well as the growing pharmaceutical, regulatory and research industry. In addition, the planned inclusion of clinical pharmacology research from multiple networks will positively impact the laboratory’s sustainability. However, it is important to note that the aforementioned will require greater capacity to accommodate an increasing number of protocols. This results in an increased number of sample analyses for more drug analytes and requires significant assay development and validation efforts.

Technologies may also change with corresponding technology advancements. Thus, continual funding, capacity building and training are crucial to keep abreast with relevant platforms.

Given the advancements in drug delivery systems such as nanoparticles and cell and tissue targeted drugs, assays in other matrices such as hair, foetal tissue and cerebrospinal fluid are desirable. Such assays require analytical systems with higher specificity and sensitivity, that is, mass spectrometry. The cost associated with acquisition, installation and training for such instrumentation is another challenge for the UZ-IPSL. To address this challenge, several strategies have been considered and are progressing. These include negotiating for allocation through new or current funding sources while pursuing collaborations within the university and other local research institutions where access to mass spectrometers might be available. Possibilities of sponsorship through philanthropic agencies are also being investigated.

## Conclusion

The UZ-IPSL provides a reproducible strategic approach for the development and implementation of an accredited PSL in a resource-limited setting. The strength of the UZ-IPSL lies in its ability to overcome several challenges through strategic partnerships, and in its diverse inter-professional human resources. These strengths should serve the UZ-IPSL well in the planned development of assays for more drugs investigated in studies.

Lessons learned

- Collaborating partnerships with local and international institutions with established laboratories were vital in overcoming implementation challenges.
- Diversity in professional specialties was key in meeting the human resource requirements for the clinical pharmacology laboratory.
